# The morphological and molecular identity of *Longidorus piceicola* Lišková, Robbins & Brown, 1997 from Romania (Nematoda, Dorylaimida)

**DOI:** 10.3897/zookeys.667.12011

**Published:** 2017-04-10

**Authors:** Mariana Groza, Stela Lazarova, Francesca De Luca, Elena Fanelli, Milka Elshishka, Georgi Radoslavov, Peter Hristov, Mihaela Coman, Vlada Peneva

**Affiliations:** 1 National Phytosanitary Laboratory, B-dul Voluntari, nr. 11 077190, Voluntari, Romania; 2 Institute of Biodiversity and Ecosystem Research, Bulgarian Academy of Sciences, 2, Yurii Gagarin Street, 1113 Sofia, Bulgaria; 3 Istituto per la Protezione Sostenibile delle Piante, Consiglio Nazionale delle Ricerche (CNR), Via Amendola 122/D, 70126, Bari, Italy

**Keywords:** D2–D3 expansion region rDNA, ITS, juvenile stages, new record, phylogeny, SNPs

## Abstract

*Longidorus piceicola*, a new geographical and host record from Romania, was described and illustrated on the basis of two populations originating from a coniferous and a deciduous forest. The main morphological characters of specimens from Romania correspond very well with the type material collected from the soil around *Picea abies* L. (Slovakia) except for the shorter body and tail. The D2-D3 fragment of 28S rDNA from both populations was amplified and sequenced, and the sequences were identical to *L. piceicola* sequence from Slovakia. The partial 18S-ITS1-5.8S-ITS2 rDNA regions from one of the populations were sequenced for the first time. The evolutionary relationships between *L. piceicola* and the closest species *L. intermedius* based on D2-D3 sequence divergence and single-nucleotide polymorphisms are discussed. Although having very low sequence dissimilarity (0.3–0.9 %) both species have distinct morphology and biology. *Longidorus piceicola* differs from *L. intermedius* in having a much longer odontostyle, body, distance anterior end - guide ring, a wider lip region, more ventromedian supplements (11 *vs* 5–7) in the male, and develops through four rather than three juvenile stages. Furthermore, *L. piceicola* occurs more frequently in association with conifers, while *L. intermedius* is found mainly in oak forests.

## Introduction

*Longidorus piceicola* Lišková, Robbins & Brown, 1997 was originally described from Slovakia (Lišková et al. 1997) in association with *Picea abies* L. Subsequently, it was recovered from different localities in Bosnia and Herzegovina, Serbia and Montenegro (Barsi and Lamberti 2001), and Poland (Kornobis and Peneva 2011) in forests dominated by coniferous trees. Here two new findings of this species in Romania are reported. The aims of this paper are to characterize morphologically and molecularly the populations recovered and to discuss the phylogenetic relationships with the most closely related species.

## Materials and methods

### Sampling and processing

Specimens were collected from the rhizosphere of a *Larix decidua* Mill. forest near to Bran, Braşov County, Romania (45.3050N, 25.2156E), ca 760 m a.s.l. on 15.10.2013, and from the soil around roots of deciduous trees (*Quercus* sp., *Tilia* sp., and *Fraxinus* sp.), Cernica forest, Ilfov County (44.2637N, 26.16514E) and ca 60 m a.s.l. on 4.08.2014. Nematodes were isolated from soil samples by a decanting and sieving technique (Cobb 1918); specimens recovered were heat killed at 55 °C for two minutes, fixed in a 4 % formalin/1 % glycerol mixture, processed to anhydrous glycerol (Seinhorst 1959), and mounted on glass microscope slides. Drawings were prepared using an Olympus BX51 compound microscope with differential interference contrast (DIC). Photographs were taken using an Axio Imager.M2-Carl Zeiss compound microscope equipped with a digital camera (ProgRes C7) and specialised software (CapturePro Software 2.8). Measurements were made using an Olympus BX41 light microscope, a digitising tablet (CalComp Drawing Board III, GTCO CalCom Peripherals, Scottsdale, AZ, USA), and computer Digitrak 1.0f programme, (Philip Smith, Scottish Crop Research Institute, Dundee, UK) and a Leica DMLB microscope with a Leica DFC 295 camera and LAS V 4.2 software.

### DNA extraction, amplification and sequencing

The genomic DNA extraction, amplification, and sequencing of single specimens of *L. piceicola* from both populations in Romania were carried out independently in two laboratories: one at the Institute for Sustainable Plant Protection, Bari, Italy and the other at the Institute of Biodiversity and Ecosystem Research, Sofia, Bulgaria. Both protocols are presented separately below.

Institute for Sustainable Plant Protection (Bari Unit): specimens (Cernica locality) for molecular analysis were kept in DESS solution (Yoder et al. 2006) before extraction. Genomic DNA was extracted from six individual female nematodes as described by De Luca et al. (2004). The crude DNA isolated from each individual nematode was directly amplified. The partial 18S-ITS1-5.8S-ITS2 regions were amplified using the forward primer TW81 (5’-GTTTCCGTAGGTGAACCTGC-3’) and the reverse primer AB28 (5’-ATATGCTTAAGTTCAGCGGGT-3’) (Subbotin et al. 2001) and the D2-D3 expansion segments of 28S rDNA was amplified using the D2A (5’-ACAAGTACCGTGAGGGAAAGTTG-3’) and D3B (5’-TCGGAAGGAACCAGCTACTA-3’) primers (De Ley et al. 1999). PCR cycling conditions used for amplification were: an initial denaturation at 94°C for 5 min, followed by 35 cycles of denaturation at 94°C for 50s, annealing at 55°C for 50s and extension at 72°C for 1 min and a final step at 72°C for 7 min. The size of amplification products was determined by comparison with the molecular weight marker ladder 100 (Fermentas, St. Leon-Rot, Germany) following electrophoresis of 10 ml on a 1 % agarose gel. PCR products of the ITS and D2-D3 regions were purified for cloning and sequencing using the protocol provided by the manufacturer (High Pure PCR elution kit, Roche, Germany). Purified ITS fragments were cloned in TA cloning vector (Invitrogen) and several clones were sequenced using an ABI Prism 377 sequencer (PE Applied Biosystem, Foster City, CA).

Institute of Biodiversity and Ecosystem Research: Genomic DNA was extracted from two single female worms *L. piceicola* from Bran locality using a standard nematode digestion protocol (Holterman et al. 2006). The D2–D3 expansion segments of the 28S rRNA gene were amplified using the same primers D2A and D3B (De Ley et al. 1999). Each PCR reaction was performed under the following conditions: initial denaturation 94°C for 5 min; 40 cycles (denaturation 94°C for 30 secs; primer annealing 50°C for 30 secs; extension 72°C for 1 min), and final extension 72°C for 10 min. For further details, see Nedelchev et al. (2014). The amplified products were sequenced by Eurofins MWG Operon, Germany.

### Sequence and phylogenetic analysis

The sequences of the *L. piceicola* have been deposited in GenBank with the following accession numbers: KY086070 and LT669801 for D2-D3 expansion domains of 28S rRNA gene; LT669802 and LT669803 for the ITS region. The D2-D3 and ITS sequences were compared with those of other nematode species available at the GenBank sequence database using BLASTN similarity search tool revealing similar results for both regions. The closest D2-D3 sequences to *L. piceicola* were aligned using ClustalX 2.1 (Larkin et al. 2007). The estimates of evolutionary divergence between the sequences of *L. piceicola* and *L. intermedius* Kozlowska & Seinhorst, 1979 (numbers of base differences and p-distances) and Single Nucleotide Polymorphism (SNP) variations (six transitions and four transversions) were performed with MEGA7 (Kumar et al. 2016). Furthermore, sequences revealing the highest similarity to *L. piceicola* were used for phylogenetic analyses; however only a midpoint rooted tree based on a reduced number of sequences (26) comprising several related species was presented here. The multiple sequence alignments used for phylogeny reconstructions were carried out using GUIDANCE2 Server (http://guidance.tau.ac.il) with the default settings (Sela et al. 2015). Bayesian Inference (BI) algorithm implemented in MrBayes 3.2.5 was used for phylogenetic relationships reconstruction (Huelsenbeck and Ronquist 2001; Ronquist et al. 2012). For further details, see Lazarova et al. (2016).

## Results

### Longidorus piceicola

Taxon classificationAnimaliaDorylaimidaLongidoridae

Lišková, Robbins & Brown, 1997

#### Material examined.

Eleven females and 21 juveniles, two females and one juvenile from Cernica forest, Ilfov County, Romania on slide numbers NE 35–37 stored at the reference collection of the National Phytosanitary Laboratory, Voluntari, Romania, 9 females and 20 juveniles - at the personal collection of the first author; nine females and 30 juveniles from Bran, Braşov County, Romania, stored in the nematode collection of IBER, Bulgaria, slide numbers N2-29/2/1-19.

#### Description.

Figures 1–7.

*Measurements* See Tables 1–3.

*Females* (Figs 1A, B1–4, G2–4, 5E, 6E, J, O, 7) based on the *Larix* population, Bran, Braşov County.

**Figure 1.**
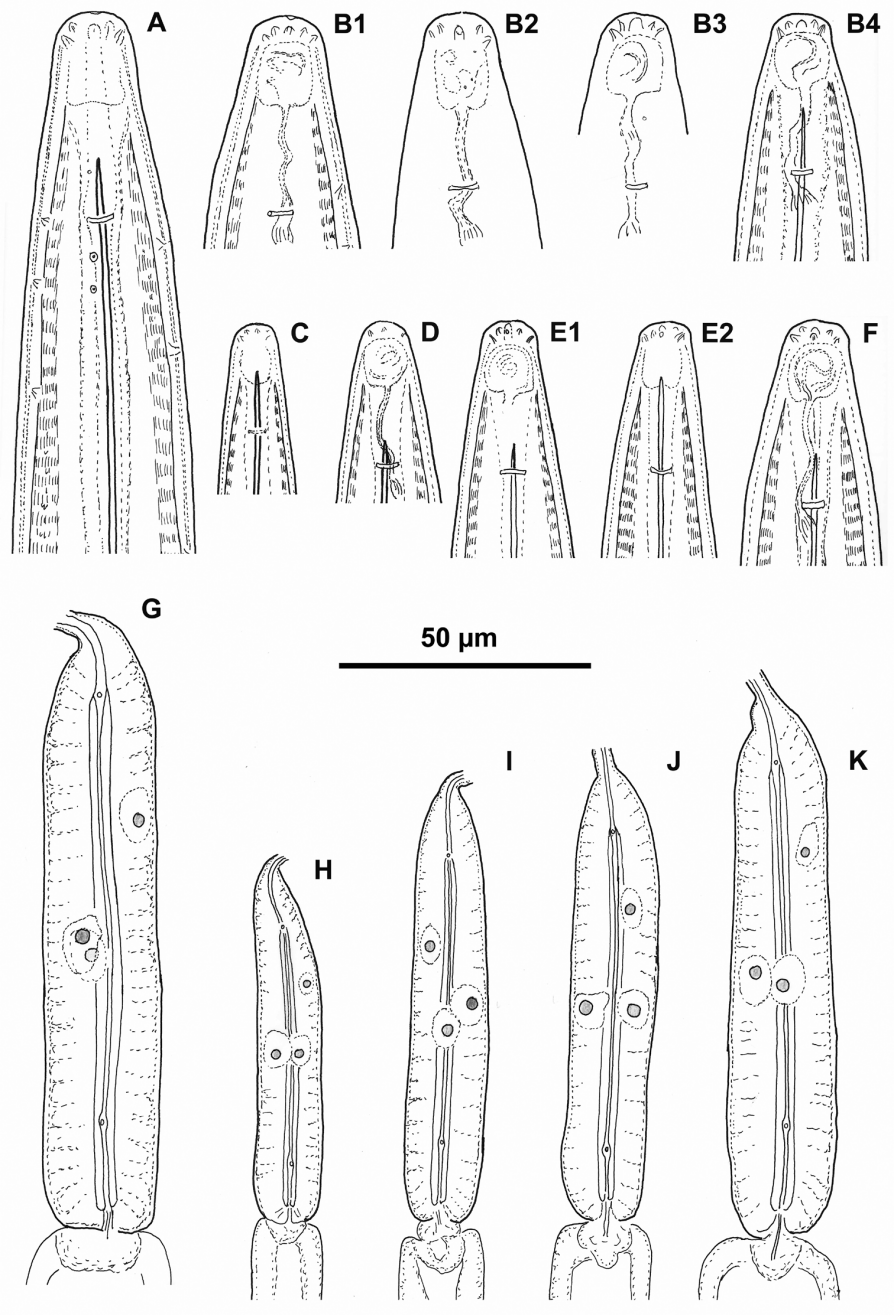
*Longidorus piceicola* Female and juveniles: **A** Neck region – female **B1–B4, C** Head end with amphidial fovea **B1–B3** females, **B4** juvenile 4^th^ stage (**B2** right and **B3** left) **C, D, E1, E2, F** Anterior ends of first- to fourth-stage juveniles **G–K** Pharyngeal bulb of female (**G**) and first- to fourth-stage juveniles (**H–K**).

Habitus spiral shaped, more strongly coiled in posterior part of body. Cuticle 3–4 μm thick at guide ring region, *ca* 3 μm in mid-body, and 5–6 μm on tail posterior to anus. Lip region broadly rounded anteriorly, rounded laterally, almost continuous with rest of body. Amphideal fovea pocket-shaped, varying from not lobed to symmetrically bilobed at base (according to terminology proposed by Decraemer and Coomans 2007) extending to *ca* half the distance anterior end-guide ring. Left and right fovea of about equal size (12.7 (11–14) μm, n = 5), sensillar pouch (fusus) just posterior the guide ring, the distance from the fovea to fusus 24 (23–29 μm). Pharyngeal bulb occupying 25 (22–29) % of total pharynx length; dorsal nucleus located at 29.5 (27–32) % (n = 7) of bulb length; ventro-sublateral nuclei at 54 (48–57) % (n = 8) (left) and 54 (52–56.5) % (n = 8) (right); opening of the dorsal gland at 9 (7.5–11) % and opening of the ventro-sublateral glands at 84 (80.5–90.5) % of the distance from anterior end of pharyngeal bulb, respectively. In one female, a small vestigium (5 μm) observed in wall of slender pharynx. Two nerve rings observed, the first one at 207.2 ± 8.8 (193–218) μm from anterior end, surrounding about mid-odontophore; the second at 329 ± 11.6 (313–344) μm from anterior end, n = 6, (first at 235.7 ± 12.7 (215–255) and second at 329.3 ± 18.6 (290–343) μm from anterior end, n = 7, Cernica forest). Tail bluntly conical, dorsally convex, flat or shallowly concave ventrally. Two pairs of caudal pores. Reproductive system didelphic, two branches of about equal size. Vagina occupies *ca* 50 % of corresponding body width; *pars distalis vaginae* and *pars proximalis vaginae* 13–15 μm and 15–19 μm long, respectively. Uteri short, anterior uterus 96.3 ± 13.5 (80–120) μm long, posterior 91.0 ± 10.5 (76–107) μm. Uteri shorter in Cernica population – anterior uterus 80.9 ± 7.0 (70–90) μm long and posterior 78.3 ± 8.3 (70–95) μm long. Sphincter between uterus and *pars dilatata oviductus* well developed. Sperm observed in both uteri of one female.

**Figure 2.**
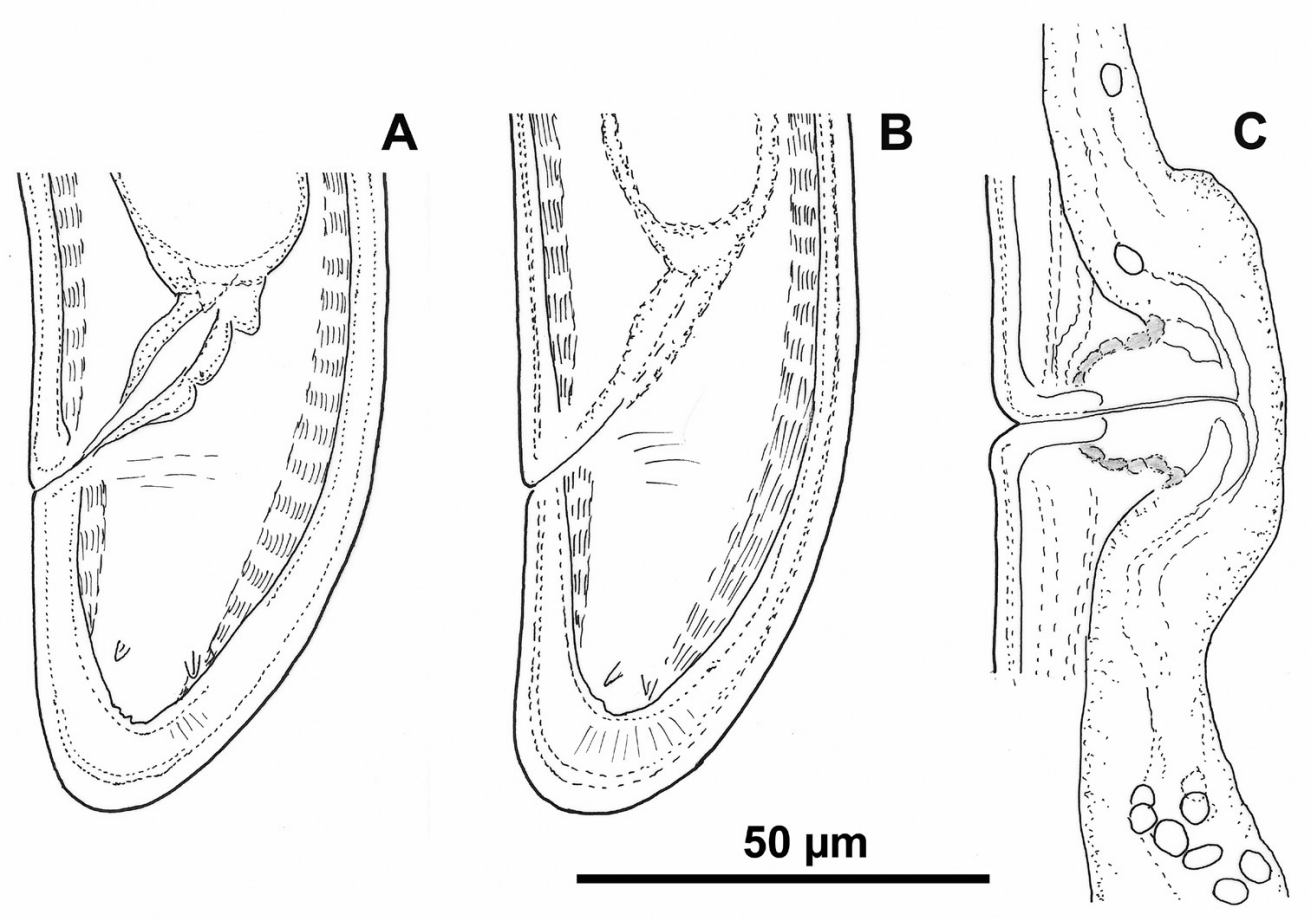
*Longidorus piceicola* Female from Bran locality: **A, B** Variations in tail shape **C** Vagina.

In the population from Cernica forest two females with reserve odontostyles have been observed (Table 3).

*Male.* Not found.

*Juveniles* (Figs 1C–F, H–K; 6A–D, F–I, K–N, 7).

General morphology similar to adult females. Body habitus similar in all stages, open C- to J-shaped. Tail of all juvenile stages conical, but becoming more rounded and c’ decreasing in subsequent stages: tail of first stage juvenile elongate conoid with slightly digitate terminus, in the second stage – elongate conoid, in third – bluntly conoid, variable, with narrow to widely rounded terminus, in fourth – resembling that of female, bluntly conoid (Fig. 5). In several juveniles, the abnormalities in their development did not allow to assign them to a particular stage and the morphometrics are presented separately (Table 3). The lengths of functional and replacement odontostyles used to infer the developmental stages were in contradiction with other measurements such as L, a, b, c etc. which were in correspondence with a different stage, or the functional odontostyle was in the ranges of one stage while the replacement one was not in the ranges of the next stage; in one occasion the length of replacement odontostyle was less than that of the replacement one (Table 3).

**Figure 3.**
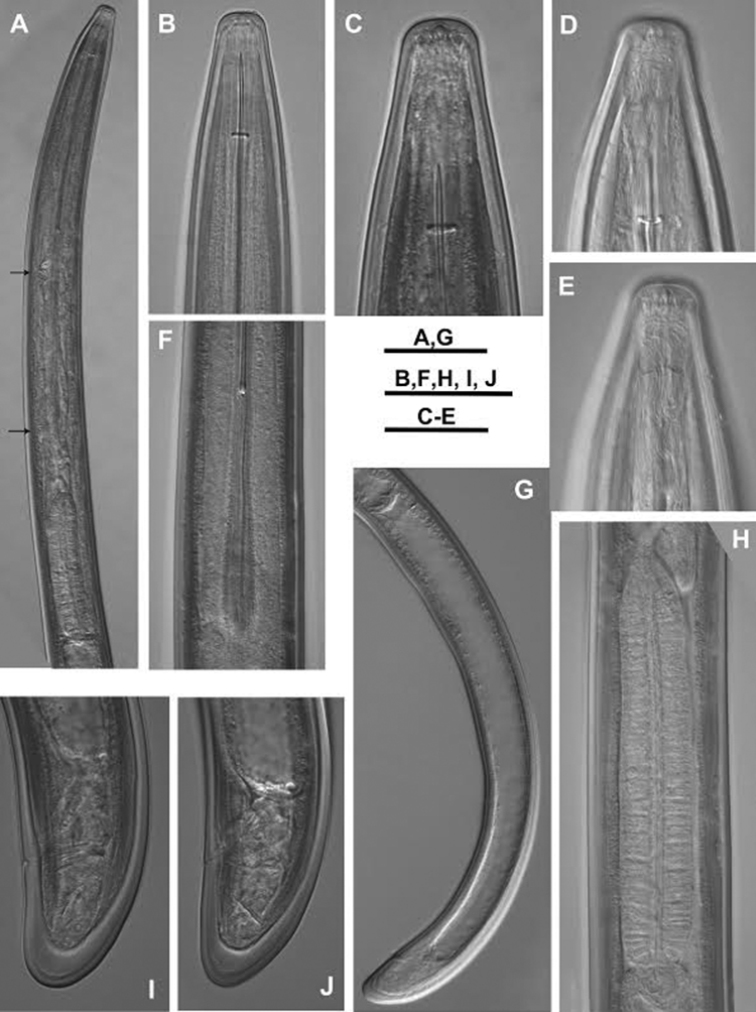
*Longidorus piceicola* Female from Bran locality: **A** Neck region, black arrows indicate nerve rings **B, C** Head end (different magnifications) **D, E** Amphideal fovea (right and left) **F** Odontophore **G** Prerectum **H** Pharyngeal bulb **I, J** Variations in tail shape. Scale bars: **A, G** 80 μm; **B, F, H, I, J** 40 μm; **C–E** 20 μm.

**Figure 4.**
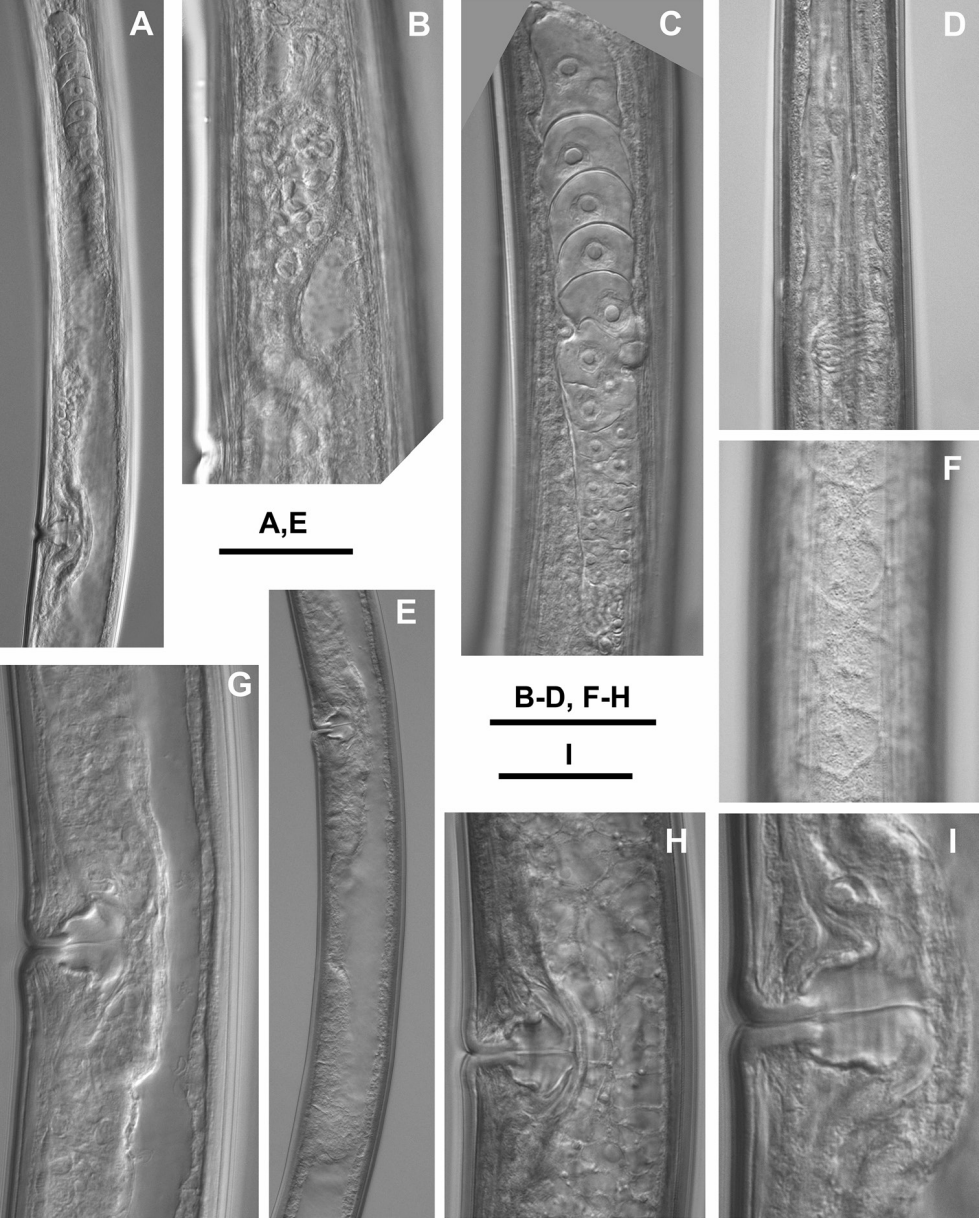
*Longidorus piceicola* Female from Bran locality: **A** Anterior genital branch **B** Uterus part with sperm **C** Ovary **D** Nerve ring **E** Posterior genital branch **F** Lateral field and epidermal glans **G–I** Variations in vagina (different magnifications). Scale bars: **A, E** 80 μm; **B–D, F–H** 40 μm; **I** 20 μm.

**Table 1.** Measurements of females and juveniles (J) of *Longidorus piceicola* from Bran, Braşov County, Romania (mean ± standard deviation, with range). All measurements in micrometers except for body length (mm).

Character	Females	J1	J2	J3	J4
n	9	6	4	8	3
L	4.90±0.47 4.05–5.64	1.32±0.11 1.15–1.47	1.83±0.16 1.63–2.02	2.62±0.13 2.38–2.81	3.21, 3.91, 3.22
a	84.6±8.0 71.1–97.3	55.4±4.6 47–60.8	59.3±5.5 53.5–65.9	67.6±3.6 62.3–71.9	73.6, 67.9, 75.5
b	9.9 ± 0.6 9.7–11.1	4.3±0.2 4.1–4.5	5.2±0.3 4.8–5.5	6.4±0.5 5.8–7.3	6.7, 8.1, 7.1
c	129.7±13.2 102.4–147.3	29.3±2.5 26.4–32.1	42.3±4.9 35–45.3	61.7±5.7 53.5–69.5	77.9, 108.4, 84.7
c’	0.97±0.06 0.89–1.10	2.8±0.3 2.6–3.2	1.9±0.2 1.64–2.15	1.45±0.1 1.38–1.58	1.23, 0.90, 1.18
V (%)	49.2±1.2 47.2–51.3	–	–	–	–
G1 (%)	6.7±0.7 5.8–7.8	–	–	–	–
G2 (%)	6.1±0.9 5.4–7.5	–	–	–	–
Developing gonad	–	16.2±1.2 15–17	28.3±7.2 20–33	33.3±2.1 31.5–37	–, 48, 45
d	2.63±0.1 2.45–2.8	2.6±0.2 2.5–2.7	2.7±0.3 2.5–3.0	2.37±1.0 2.5–2.8	2.9, 2.8, 2.9
d’	2.02±0.1 1.9–2.1	1.8±0.1 1.65–2.3	1.95±0.25 1.7–2.3	1.9±0.1 1.7–1.9	2.0, 2.1, 2.1
Odontostyle	155.5±5.2 147–163	95.8±1.2 82–90.3	100.7±3.0 97.5–105	118.4±3.7 115–125	130, 143, 142
Replacement odontostyle	–	103.7±3.5 99.5–110	115.4±6.0 109–123	137.8±2.7 134–143	151, 153, 154
Odontophore	77.7±3.4 71–82	47.5±1.4 46–50	55±4.2 50–60	62.9±2.9 60–68	75, 73, 73
Anterior end to guide ring	38.1±1.9 35–41	22.0±1.3 22–24	26±1.1 25–27	29.9±1.7 27–33	36, 37, 35
Bulbus length	118.5±7.9 105–130	65.9±4.5 59–69	71.8±3.4 75–83	91.1±3.6 86–97	104, 116, 101
Bulbus width	23.4±1.8 20–25	13.8±1.2 13–14	16.6±0.5 16–17	19.2±0.6 18–20	22, 22, 21
Pharynx	478.4±29.4 440.5 –528	307.6±12.3 290–319	352±12.9 338.5–364	409.3±22.9 374–447	480, 484, 455
Tail	38.2±1.8 35 – 42	45.4±4.2 42–51.5	43.5±2.9 40.5–47	42.7±4.2 36–48	41, 36, 38
Length of hyaline part	11.7±0.9 10–13	9.5±0.6 9–10	8.5±0.6 8–9	9.3±1.2 8–11	9.5, 12, 8
Body diameter at: – lip region	14.5±0.6 14–16	8.6±0.6 8–10	9.6±0.6 9–10	11.1±0.3 11–12	12, 14, –
– guide ring	29.2±1.6 27–32	15.3±0.7 14.5–16	18.5±1.3 28–31	21.1±1.2 19–23	25, 29, 26
– base of pharynx	48.4±3.3 44–55	22.8±0.6 23–24	29.2±1.3 28–31	36.2±2.3 32–40	39,47, 39
– mid–body/at vulva	58.7±5.4 53–71	23.8±0.8 23–25	30.9±1.8 29–33	38.9±2.7 33–41.5	44, 58, 43
– anus	39.7±3.5 35–46	16.1±0.6 15.5–17	23±1.6 22–25	29.5±2.4 25–32	34, 37, 30
– hyaline part	24.9±3.5 18–29	7.4±0.6 6.7–8.4	10.8±0.3 10.5–11	16.1±1.5 14–18	–, 25, 18

d, distance from the anterior end / body diameter at lip region. d’, body diameter at guide ring / body diameter at lip region (Brown et al., 1994).

**Table 2.** Measurements of females and juvenile stages (J) of *Longidorus piceicola* from Cernica-Ilfov County, Romania (mean ± standard deviation, with range). All measurements in micrometers except body length (mm).

Character	Females	J1	J2	J3	J4
n	9	11	2	3	5
L	5.88±0.19 5.17–6.54	1.36±0.09 1.21–1.52	1.79, 2.16	3.29, 3.04, 2.81	3.95±0.47 3.6–4.7
a	95.2±11.5 73.8–105.5	58.96±4.9 53–66.8	64.1, 67.7	68.7, 67.7, 68.7	77±8.2 62.5–83.4
b	10.2±1.2 8.4–12.7	4.88±0.8 4.1–6.7		8.4, 9, 6.6	9±1.5 6.9–10.6
c	171.9±28.8 134.4–218.0	31.0±1.9 28.3–33.4	–	79, 71,64.3	102±8.1 89.8–109.8
c’	0.85±0.10 0.72–0.99	2.9±0.2 2.6–3.1	–	1.3, 1, 1.6	1±0.1 0.9–1.2
V (%)	48.1±0.98 47.1–50.6	–		–	–
G1 (%)	5.8±0.8 4.7–7.1	–	–	–	–
G2 (%)	5.4±0.7 4.4–6.4	–	–	–	–
d	2.9±0.1 2.7–3.1	2.9±0.2 2.6–3.3	2.78, 2.99	3, 3, 3.4	3±0.2 2.8–3.2
d’	1.8±0.1 1.7–1.9	1.9±0.2 1.5–2.2	1.8, 1.7	2, 2, 2	2±0.1 1.7–2
Anterior end to guide ring	42.2±1.8 40–45	22.8±1.4 21–26	25, 29	34.8, 34, 34	37.2±0.9 36–39
Odontostyle	155.4±5.4 150–165	86.9±2.7 82–90	97, 102	122, 124, 108	136.8±3.4 132–141.5
Bulbus length	135±4.9 126–141	72.7±4.3 65–78.5	71, 86	104, 104, 100	113.7±5.9 108–120
Bulbus width	24.7±2.0 22–29	12.5±0.9 11–14	15, 14.5	18, 21, 19	19.9±2.1 17–21
Replacement odontostyle	–	95.7±3.7 92–102	109, 111	142, 136, 132	157.3±6.7 150–165
Odontophore	78.1±4.9 70–83	52.5±4.8 48–65	55, 60	72, 60, 65	72.2±3.0 69–76
Oesophagus length	579.3±47.6 514–661	284.9±42.4 219–356	320, 366	393, 321,425	460.3±84.3 345–545
Tail	34.8±4.4 30–41.5	43.58±2.3 39–47	–	42, 43, 44	38.7±3.5 34–43
Length of hyaline part	12.3±1.1 11–14	9.5±0.9 8–11	10, 10	9, 10, 10	10±0.6 9.4–11.1
Body diameter at: – lip region	14.7±0.4 14–15	7.9±0.2 7.5–8	9, 10	11, 11,10	12.4±0.5 12–13
– guide ring	26.1±1.2 24–27	14.7±1.5 12–18	16, 16	20.5, 21, 20	23.16±0.6 22.5–24
– base of pharynx	53.6±6.1 45–62	21.9±0.9 20–23	25, 28.6	39, 38, 38	43.5±3.0 38.9–46.4
– mid–body/at vulva	63.2±4.5 58–70	23.2±1.4 21–26	28, 32	48, 45, 41	51.8±5.8 45–58
– anus	40.8±2.1 38–44	15.2±1.3 14–18	–	31, 30, 27	36±3.4 33.2–41.2
– hyaline part	27.8±1.9 25–31.5	7.4±0.6 7–8	7, 8	18, 16, 15	22±1.4 20–23

**Table 3.** Measurements of *Longidorus piceicola* females (f) from Cernica, and juveniles (j) from Bran, Braşov County, Romania showing different anomalies. All measurements in micrometers except body length (mm).

Character	f	f	j	j	j	j	j	j	j	j
No	1	2	1	2	3	4	5	6	7	8
L	5.95	5.86	4.73	2.34	2.72	2.71	2.62	1.14	3.66	2.71
a	99.1	97.7	93.0	63.6	77.7	61.9	67.0	32.6	75.1	63.8
b	9.5		10.3	5.6	6.0	5.9	7.1	2.9	7.9	6.2
c	220.3	172.4	98.8	60.5	65.2	61.9	61.4		–	70.3
c’	0.75	0.94	1.3	1.3	1.6	1.4	1.7		–	1.2
V	49.2	48.9	–	–	–	–	–	–	–	–
Developing gonade	–	–	65	–	–	–	22		27	
d	2.93	2.73	2.6	2.8	2.9	2.9	2.5		2.7	2.8
d’	1.79	1.80	1.4	2.0	2.0	2.0	1.8		1.9	1.8
Odontostyle	165	158	117	127	122	105	81	106	120	125
Replacement odontostyle	175	158	131	165	165	135	108	130	140	156
Odontophore	80	70	78.5	61	65	65	60		60	73
Anterior end to guide ring	41	41	35	30	32	33	25	26	32	32
Bulbus length	132	130	114	81	87	89	95	93	108	86
Bulbus width	23	23	22	19	18	20	17			
Pharynx	627	–	461	420	457	464	369	387	463	441
Tail	27	34	48	39	42	44	43		–	39
Length of hyaline part	11	8	10	9	9	6	9			
Body diameter at: - lip region	14	15	14	11	11	11			9	11.5
- guide ring	25	27	19.5	21	22	23	11		19	21
- base of pharynx	51	50	42	35	32	38	32	29	40	32
- mid-body/at vulva	60		51	37	35	44	39	35	49	43
- anus	60		38	29	26	32	24		–	32
- hyaline part	25	23	20	16	14	15			11	18

#### Sequences and phylogenetic analyses.

The amplification of the ITS and the D2-D3 expansion domains of the 28S rRNA gene yielded fragments of 1646 and 756 bps, respectively, based on sequencing. The ITS sequences of *L. piceicola* from Romania were obtained for the first time in the present study. They showed 98 % similarity (962/984 identities, 9 gaps) when compared with the corresponding sequence of *L. intermedius* (KT308890) and 86 % with the ITS sequence of *L. elongatus* Hooper, 1961 (AJ549986, AJ549987). Intraspecific variation for the ITS sequences was low, with only two nucleotides difference and no indels.

**Figure 5.**
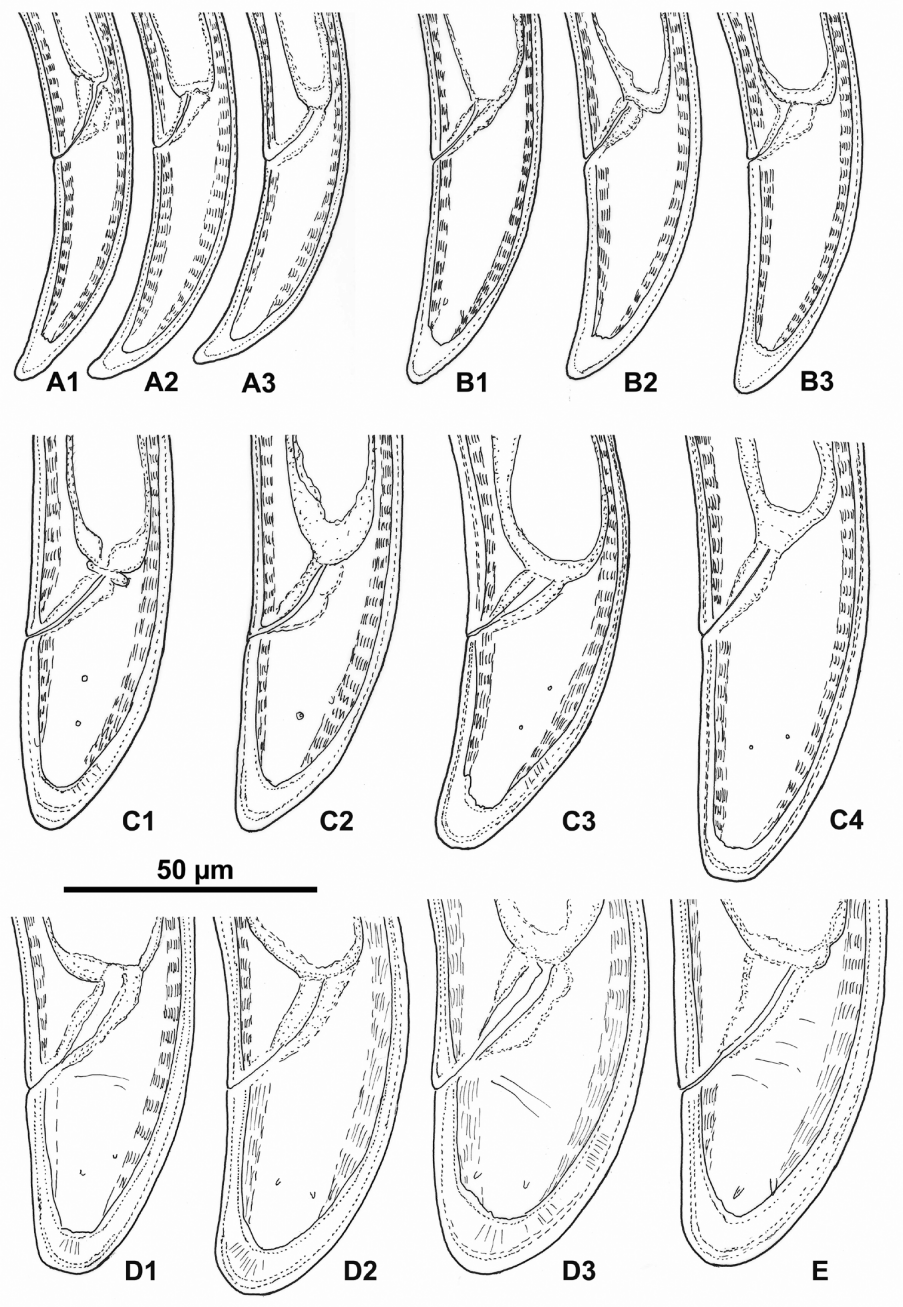
*Longidorus piceicola* Juveniles and female from Bran locality: Variations in tail shape of first (**A1–A3**), second (**B1–B3**), third (**C1–C4**), fourth (**D1–D3**) juvenile stages and female (**E**).

D2-D3 rDNA sequences obtained from both Romanian populations were identical to each other and to the sequence of *L. piceicola* from Slovakia (AY601577, He et al. 2005). The phylogenetic relationships of *L. piceicola* with several related species is presented in Figure 8. *Longidorus intermedius* revealed sister relationships with *L. piceicola* and the sequences from both species formed a well-supported clade. In addition, five sequences of *L. intermedius* from Germany (AF480074, Rubtsova et al. 2001), Russia (KF242311 and KF242312, Subbotin et al. 2014), Spain (KT308868, Gutiérrez-Gutiérrez et al 2013 and JX445117, Archidona-Yuste et al. 2016), and the *L. piceicola* sequence were realigned separately and pairwise distances estimated. A total of 737 positions was included in the dataset. The between species dissimilarities (p-distances) were 0.3–0.9 % (or 2–6 bp differences). Similarly, the intraspecific p-distances of *L. intermedius* from the three European countries were 0.4-0.9 % (i.e. 3–6 bp).

**Figure 6.**
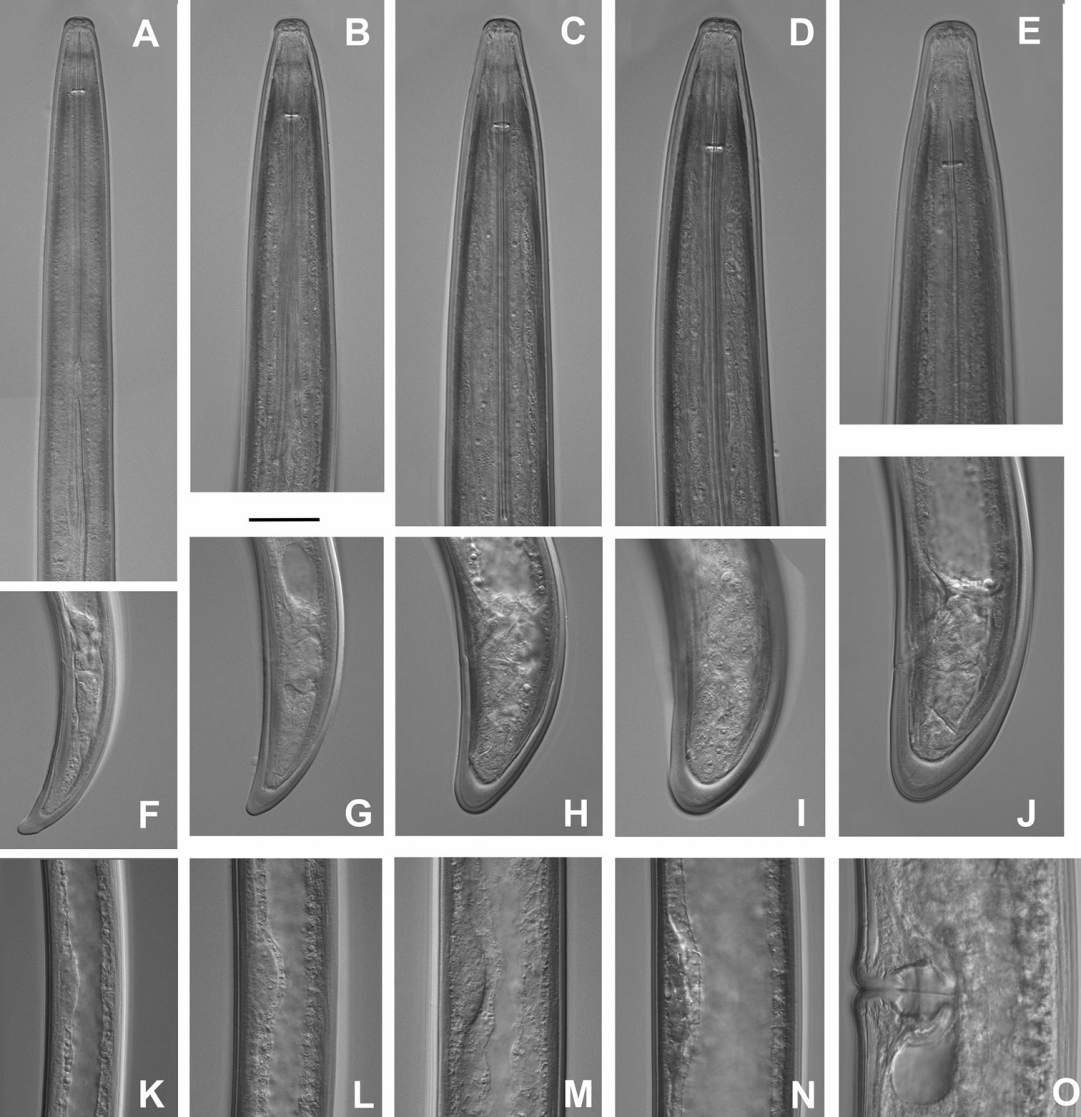
*Longidorus piceicola* Juveniles and female from Bran locality: **A–E** Anterior ends of first- to fourth-stage juveniles and female **F–J** Tails of first to fourth juvenile stages and female **K–M** Genital primordium of first to fourth juvenile stages. **O** Vagina. Scale bar: 20 μm.

The SNPs analysis comparing all D2-D3 sequences of *L. piceicola* and *L. intermedius* revealed three parsimony-informative sites (i.e. nucleotide positions with transitions 89T/C, 134T/C and 297A/G) when compared to the reference sequence of *L. piceicola* (AY601577) (Table 4). The most similar sequence to the *L. piceicola* sequence was that of *L. intermedius* from Germany, revealing the highest similarity and only two interspecies differentiating nucleotides at positions 89 and 134 compared to the reference sequence (Table 4).

**Figure 7.**
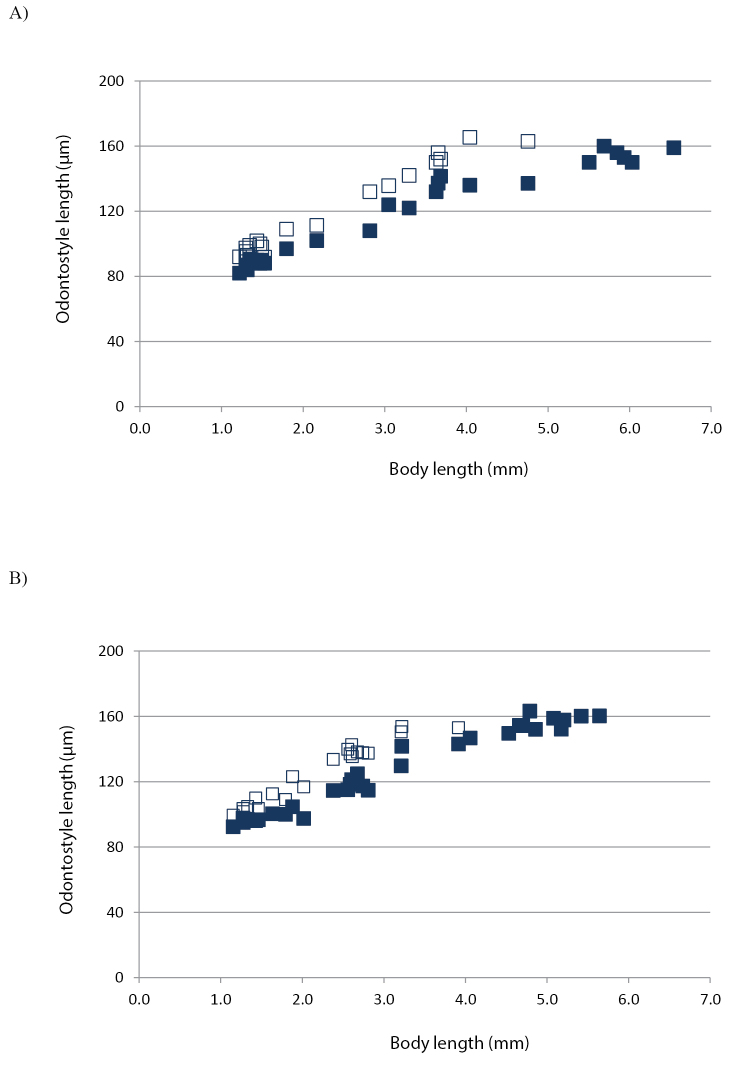
Scatter plot of odontostyle (■) and replacement odontostyle (□) against body length of *Longidorus piceicola* juveniles (J1 to J4) and females from **A** Cernica forest, Ilfov county and **B** Bran locality, Braşov county.

**Figure 8.**
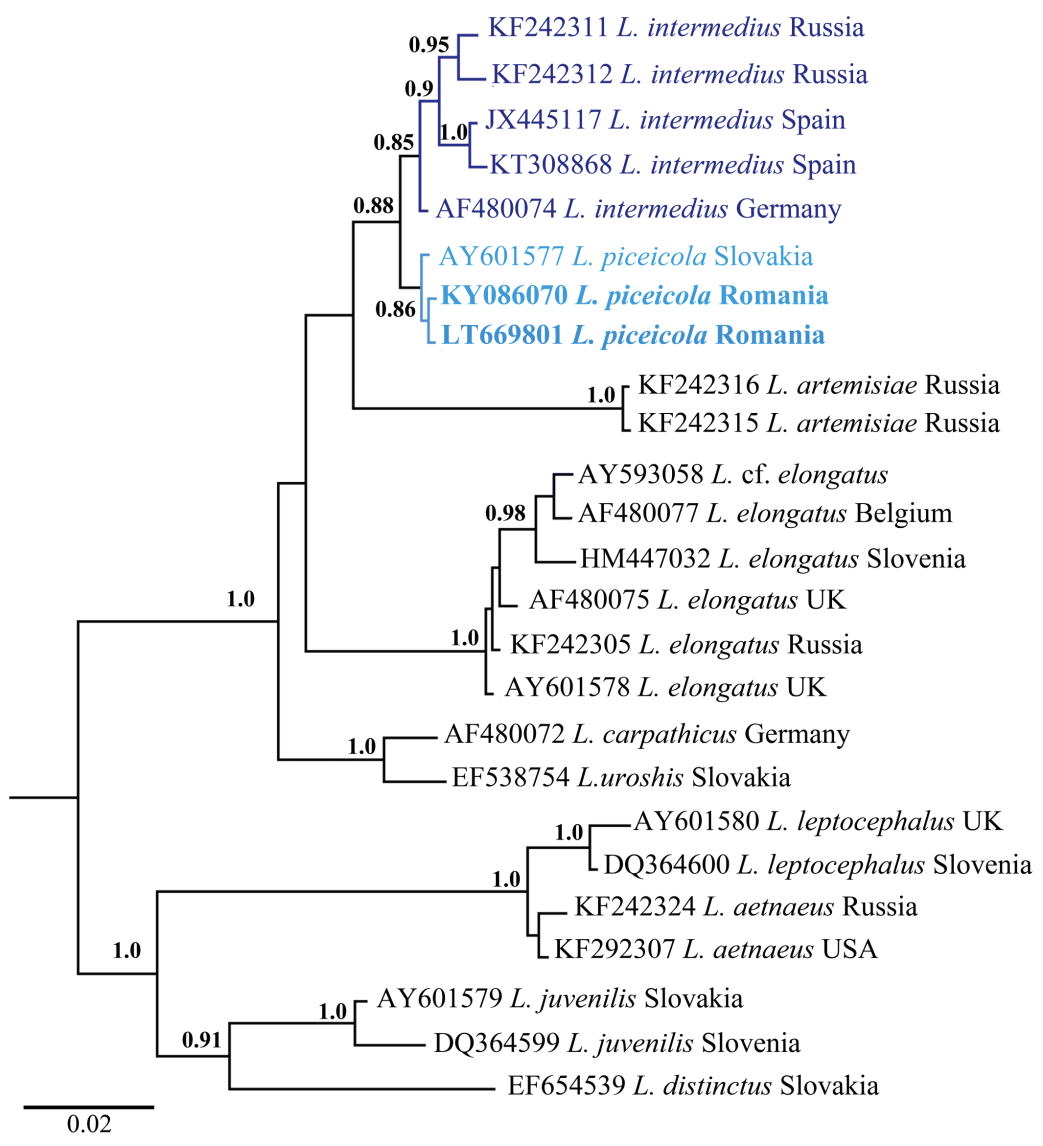
Phylogenetic tree using D2-D3 28S rDNA and inferred from a Bayesian analysis with GTR+G model and midpoint rooting. Posterior probabilities ≥ than 0.8 are presented.

**Table 4.** The variable positions in D2-D3 28S rDNA control region sequences of *Longidorus piceicola* and *L. intermedius.* The *L. piceicola* sequence from Slovakia (Acc. no AY601577) was used as a reference.

	SNPositions											
	89	129	134	197	255	285+1gap	285+2gap	297	310	413	514	584
**AY601577 reference sequence**	**T**	**C**	**T**	**A**	**C**	–	–	**A**	**T**	**G**	**G**	**C**
AY601577 *L. piceicola* Slovakia	.	.	.	.	.	–	–	.	.	.	.	.
KY086070 *L. piceicola* Romania 1	.	.	.	.	.	–	–	.	.	.	.	.
LT669801 *L. piceicola* Romania 2	.	.	.	.	.	–	–	.	.	.	.	.
AF480074 *L. intermedius* Germany	**C**	.	**C**	.	.	–	–	.	.	.	.	.
JX445117 *L. intermedius* Spain	**C**	.	**C**	.	T	A	T	**G**	G	.	S	.
KT308868 *L. intermedius* Spain	**C**	.	**C**	.	T	A	T	**G**	G	.	T	.
KF242312 *L. intermedius* Russia	**C**	T	**C**	.	.	–	–	**G**	.	T	.	T
KF242311 *L. intermedius* Russia	**C**	.	**C**	C	.	–	–	**G**	.	T	.	.

## Discussion

Morphologically, the specimens of *L. piceicola* from Romania are similar to the type-population from Slovakia (Lišková et al. 1997), except for the slightly longer body (av. 5.88 *vs* 5.19 mm) and shorter tail (av. 34.5 *vs* 42 µm, av. *c* = 172 *vs c* = 125) in the population from Cernica forest. Barsi and Lamberti (2001) described several *L. piceicola* populations from Bosnia and Herzegovina, Serbia and Montenegro. In comparison with those populations, the nematodes from Romania have a narrower lip region (avs. 14.5, 14.7 *vs* avs. 16–17 µm), a shorter odontostyle (avs. 155.4, 155.5 *vs* avs. 167–188 µm) and tail (av. 35 *vs* avs. 39–46 μm) in specimens from Bran population. Compared to subsequently recorded *L. piceicola* population from Poland, specimens from Romania have, again, a much shorter body (avs. 4.9, 5.2 *vs* av. 6.5 mm) and tail (avs. 34, 38 *vs* av. 47.4 μm).

The observed abnormalities (presence of reserve odontostyle) in females have been reported for other longidorids (Ferris et al. 2012) whereas atypical development in juveniles has not been recorded previously to such a great extent (*ca* 30 % of all juveniles studied from *L. decidua* forest were atypical). Ferris et al. (2012) hypothesized that “anatomical aberrations possibly are results from accidents in transcription of the genetic code or mutations which may or may not be mechanistically limiting to reproduction and therefore may or may not be maintained in the genome through either apomixis or amphimixis”.

*Longidorus piceicola* was previously recovered in association with *P. abies*, *Abies alba* L., *Fagus sylvatica* L., *Carpinus betulus* L. and *Vitis vinifera* L. in Slovakia, West Balkans and Poland (Lišková et al. 1997, Barsi and Lamberti 2001, Kornobis and Peneva 2011, Skwiercz et al. 2015), and our findings in coniferous forest dominated by larch and mixed deciduous forest (*Fraxinus*, *Quercus* and *Tilia*) in Romania extend the geographical and plant association ranges further southeast.

Based on the molecular and morphological characterization *L. piceicola* is closely related to *L. intermedius*: however, it differs in having a much longer odontostyle (151–169 μm in the type population and reported range of 144–183 μm *vs* 105–118 μm and 97–121 μm, respectively), generally longer body (4.22–5.97 mm in the type population and reported range of 4.42–7.99 mm *vs* 3.6–4.5 mm and 3.11–5.4 mm, respectively) and bigger anterior end – guide ring distance (37–45 μm in the type population and a range of 34–46 μm *vs* 25–34 μm and 27–36 μm, respectively); a wider lip region (14–18 *vs* 11–12.5 μm), more ventromedian supplements (11 *vs* 5–7) in the males, and four *vs* three juvenile stages (Lišková et al. 1997, Peneva et al. 2001, Barsi and Lamberti 2001, Kumari et al. 2006, Kornobis and Peneva 2011, Gutiérrez-Gutiérrez et al. 2013). Sequence and SNPs analyses of the D2-D3 rDNA region of *L. piceicola* and *L. intermedius* populations showed three transitions and four transversions that can be used to s differentiate between both species. Furthermore, *L. piceicola* was more frequently found in association with conifers, while *L. intermedius* occurred mainly in oak forests.

## Supplementary Material

XML Treatment for Longidorus piceicola
